# Laboratory Exposures to Brucellae and Implications for Bioterrorism

**DOI:** 10.3201/eid1108.041197

**Published:** 2005-08

**Authors:** Pablo Yagupsky, Ellen Jo Baron

**Affiliations:** * Ben-Gurion University of the Negev, Beer-Sheva, Israel;; † Stanford University Medical School, Stanford, California, USA; +This page was updated on October 7, 2005 to incorporate the corrections in Vol. 11, No. 11

**Keywords:** laboratory-acquired brucellosis, prophylaxis, bioterrorism, exposure

## Abstract

Brucellae are class 3 organisms and potential agents of bioterrorism. Because of effective public health measures, brucellosis has become a rare disease in industrialized countries, and clinical microbiology laboratories are frequently unfamiliar with the genus. A low index of suspicion by physicians or failure to notify the laboratory, equivocal Gram-stain results, misidentification of the organism by commercial systems, unsafe laboratory practices, and laboratory accidents have been responsible for numerous cases of exposure to the organism and laboratory-acquired disease in recent years. Discovery of a laboratory exposure to brucellae should prompt an exhaustive investigation of the event and its circumstances, definition of the population at risk, enforcement of safe laboratory practices, and antimicrobial drug prophylaxis for exposed persons. Inadvertent exposures to brucellae in the clinical laboratory indicate a widespread lack of preparedness to cope with eventual biologic threats involving use of the organism.

## Abstract 2

Laboratory exposures to brucellae indicate unfamiliarity with organism.

Brucellosis is a zoonosis usually transmitted to humans by contact with infected animals and consumption of contaminated animal products (1,2). Because of compulsory pasteurization of milk products and strict control of the disease in dairy cattle, the incidence of brucellosis has steadily declined in most industrialized countries during the last 50 years. However, the disease remains among the most commonly recognized causes of laboratory-transmitted infections; 2% of all brucellosis cases are laboratory-acquired (1,3–9).

## The Organism

Several biologic characteristics make brucellae easily transmissible within the close confinement of the clinical microbiology laboratory, including the facts that the infecting dose for humans is low, and the organism may enter the body in many ways relevant to laboratory practices (e.g., through the respiratory mucosa, conjunctivae, gastrointestinal tract, or abraded skin) (1,2). Rare cases of acquisition of the organism through organ transplantation, sexual contact, breastfeeding, or the transplacental route have also been reported (1). Because person-to-person transmission does not occur, infected persons do not pose a threat to their surroundings.

Soon after entry into the body through the skin or mucous membranes, brucellae are ingested by polymorphonuclear and mononuclear phagocytes. The organism is able to escape phagocytic killing by inhibiting the phagosome-lysosome fusion and reproducing inside macrophages (1,10). After a variable incubation period ranging from <1 week to several months (usually 2–4 weeks), nonspecific systemic symptoms such as fever, headache, malaise, night sweats, and arthralgia follow, resembling a flulike disease (1,2). During the early stages of the disease, patients are frequently bacteremic. This bacteremia has a continuous pattern, making circulating brucellae easily detectable by blood culture. Once in the bloodstream, the organism is seeded to multiple organ systems and especially to those rich in reticuloendothelial tissue, such as the liver, spleen, and the skeletal and hematopoietic systems, where it may cause localized disease such as hepatitis or arthritis (1,2).

Because of the variable manifestations of human brucellosis, a wide array of different clinical specimens may contain viable brucellae, including pus, blood, bone marrow, synovial fluid and tissues, and more rarely, cerebrospinal fluid, urine, and genital exudates. The concentration of *Brucella* organisms in the blood (11,12) and synovial fluid (13) of patients with brucellosis is usually low, and therefore, these clinical specimens probably pose a low risk for contagion for laboratory personnel. However, the danger of clinically relevant exposure increases exponentially after incubation of both solid and liquid media. Seeded media harbor considerable amounts of viable *Brucella* organisms, and routine bacteriologic procedures such as preparing, centrifuging, and vigorous agitation (vortexing) of bacterial suspensions, performing subcultures and biochemical testing, and particularly the catalase test, may create dangerous aerosols and the potential for accidental spillage (14).

Most cases of laboratory-acquired brucellosis have been caused by the more virulent *Brucella melitensis* species (3,4,6,8,14–29). *B. suis* (4,6), *B. abortus* (3,6,8), and *B. canis* (30) have also been implicated, and transmission of the attenuated *B. abortus* 19 and *B. melitensis* Rev-1 vaccine strains has also been reported (31,32).

### Clinical Manifestations of Laboratory-acquired Infections

Patients involved in laboratory outbreaks of brucellosis have shown almost the entire range of clinical manifestations of the disease, ranging from the common prolonged febrile syndrome (undulant fever) (14) and a flulike disease (25,27), to focal signs and symptoms, such as hepatitis (17,33), lymphadenopathy (17,25), uveitis (14), breast abscess (28) epididymitis (29), arthritis (17), discitis (29,34), pneumonitis (17), deep vein thrombosis (29), and meningitis (31). Deaths are rare (<5%) even among untreated patients with brucellosis, but 5 fatal cases occurred among the 426 persons with laboratory-acquired disease summarized by Pike in 1978 (5).

### Laboratory Exposure to *Brucella* Organisms

Because of effective control measures in animals and animal products, brucellosis has been almost eradicated from most industrialized countries, where the disease is usually limited to persons who have traveled to developing countries or ingested imported contaminated food (14). Human brucellosis has become so rare in the United States that <300 cases have been reported annually in the last 4 decades (9,15) and ≈100 per year in the last 10 years (2). Because of the rare occurrence of brucellosis, technicians working in industrialized countries have become unfamiliar with the staining and other phenotypic characteristics of the organism. In addition, physicians frequently do not consider the diagnosis of brucellosis or fail to communicate their suspicion to the clinical microbiology laboratory, which results in inadvertent handling of cultures on an open bench.

Although the identification of the genus *Brucella* is straightforward (small gram-negative coccobacilli; positive oxidase, catalase, and urease test results; no sugar fermentation; and requirement of aerobic conditions and added CO_2_ for its growth), laboratory-acquired disease has frequently resulted from misidentification of the organism (35). In 2 published outbreaks, the *Brucella* isolate resisted decoloration and appeared as a gram-positive or gram-variable coccobacillus and thus was misidentified as micrococcus or a coryneform bacillus (14,29). In other cases, *B. melitensis* organisms tested with the API20NE (bioMérieux, Marcy l'Etoile, France) identification kit produced the 1200004 or 1000004 biochemical profile leading to misidentification of the isolate as *Moraxella phenylpyruvica* at a good identification level (90.5%) (8,18–21). The frequent failure of clinical laboratories to correctly identify isolates as brucellae is particularly worrisome because these organisms are regarded as potential agents for bioterrorism. Brucellae are inexpensive to produce and disperse, and transmission to humans may result in prolonged illness and long-term sequelae. These organisms are considered category B select agents (2). The occurrence in the last few years of inadvertent exposures to brucellae in laboratories in industrialized countries (7,8,14,17–26) indicates a lack of preparedness to deal with a real biologic threat. Lack of recognition of an isolate as a *Brucella* sp. by a laboratory may enable a bioterrorism-related attack to go undetected, whereas a false-positive identification may cause unnecessary alarm.

In laboratories serving countries in which human brucellosis is still endemic, the degree of potential exposure to the organism may be extremely high. In a microbiology laboratory in Ankara, Turkey, the average annual number of cultures positive for *Brucella* spp. reaches 400 (34). A laboratory serving a brucellosis-endemic area in southern Israel processes ≈150 per year; at the peak of the *Brucella* season (April–June), 10% of all positive blood cultures grow *B. melitensis* (27). The risk for exposure in developing countries is frequently aggravated by lack of safety equipment and inadequate laboratory facilities (34). In the aforementioned Turkish laboratory, the disease affected 10 (18%) of 55 laboratory workers, and the calculated hazard was 8% per employee-year (34).

### Mechanism of Transmission

The probable source of the infection is usually apparent when disease occurs in laboratories where isolation of brucellae is rare (21). In laboratories serving brucellosis-endemic areas, the time and circumstances of contamination are more difficult to trace because of the existence of multiple potential sources (22,27). Although the exact form of transmission often remains speculative, aerosols have been implicated in most cases (24,27,29,32). This assumption has been strengthened by spread of the disease to distant areas through common ventilation (27,32).

Laboratory accidents such as breakage of centrifuge tubes (7) or blood-culture vials (16) play a minor role in laboratory-acquired disease and are responsible for only 20% of cases (6). More commonly, exposures are the result of unsafe laboratory practices, such as sniffing plates (8,24,26,29,34); working on an open bench with viable organisms (14,16,17,20,22); not using protective equipment such as gloves, masks, and goggles (34); or ingesting suspensions of living brucellae during mouth pipetting (3). However, even when no apparent breach in safety procedures is apparent, transmission may occur (17). Rare events—such as self-inoculation of brucellae by syringes loaded with a suspension of the organism or with synovial fluid from an infected patient (3,16), injury to the conjunctiva with a broken tube that had contained a living culture (3), or participation in a laboratory exercise in which still-viable organisms were inadvertently used (15)—are also responsible for a small number of laboratory exposures and outbreaks of disease.

Most cases of laboratory-acquired brucellosis have occurred in clinical laboratories, but transmission of the disease in research facilities (4,25) and laboratories that manufactured *Brucella* vaccines has also been documented (21,22). A laboratory technician at the Centers for Disease Control and Prevention (CDC) became infected while working in a safety cabinet with a *B. melitensis* isolate that had originally caused an outbreak of laboratory-acquired disease in a community hospital (17). In a separate episode, the organism isolated from a microbiology technologist with laboratory-acquired disease infected a laboratory worker at the hospital to which the technologist had been admitted (14).

The attack rate of laboratory-associated infections has ranged from 30% to 100% depending, among other factors, on the location of workers, whether aerosol-generating procedures have been performed, and the concentration of microorganisms in the contaminated media (7,8). However, the hazard of transmission is not limited to persons who worked with the isolate. Among the 74 reported cases of laboratory-acquired brucellosis from 1897 to 1939 reviewed by Meyer and Eddie, the disease also affected janitors and occasional visitors (3). In a large outbreak of laboratory-acquired brucellosis, 3 of 7 cases of disease occurred among persons who briefly visited the facility but did not enter the room where *Brucella* cultures were processed (27).

### Investigating Outbreaks of Brucellosis

Recognizing sporadic cases and even outbreaks of laboratory-acquired brucellosis is not always easy because the disease lacks distinctive clinical features (14,17) and has a widely variable incubation period (7,17,19,32). In a cluster of disease that originated in a single exposure, the onset of symptoms of the 7 affected laboratory workers spanned 5 months (17). Although a temporal clustering of cases usually suggests a large exposure from a pinpoint source (7,32), this is not always the case. When biotyping was performed in the isolates recovered within a 5-week period from 7 infected hospital workers in southern Israel, 3 distinct strains were recognized, demonstrating that the outbreak was caused by at least 3 separate exposures (27).

### Guidelines for Investigating Outbreaks

In-depth investigation of laboratory-acquired cases of brucellosis may lead to the identification of unsafe bacteriologic practices, suitable for correction by educational and technical measures. To guide the investigation, the following recommendations are made. 1) Send isolates to a reference laboratory for confirmation. Organisms should be shipped as "dangerous goods," according to the guidelines established by the World Health Organization and the Office of Biosafety at CDC (36). In the United States, organisms presumptively identified as brucellae should be sent to CDC or another public health laboratory following the specific guidelines for transferring "select agents." 2) Inform infection control services and public health authorities, who may choose to involve CDC. 3) Conduct a meticulous epidemiologic investigation. 4) Determine the date and circumstances of the exposure (17). 5) Exclude other potential sources of transmission, such as previous laboratory exposures, travels to brucellosis-endemic areas, consumption of unpasteurized dairy products, and handling of farm or laboratory animals (14,15,17,27,29). 6) Keep all relevant data and records. 7) Define the exposed population. 8) Determine the level of risk on the basis of type of laboratory procedures performed, proximity to the source, duration of the exposure, and the like, to define persons to whom postexposure prophylaxis should be offered (8,32). 9) Check biologic safety cabinets (27,29). 10) Check the ventilation system (27). 11) Collect baseline serum samples from all known potentially exposed persons (17,27). 12) Freeze isolates for future typing, especially in brucellosis-endemic areas (23,27).

### Prevention of Laboratory-acquired Cases

Brucellae are considered class 3 organisms. CDC has strongly recommended that live *Brucella* cultures and suspicious organisms be manipulated in a class II biologic safety cabinet (37). This recommendation, however, is clearly insufficient for preventing laboratory-acquired disease because by the time the organism is suspected or confirmed as *Brucella*, exposure of laboratory personnel may have occurred (27). On the other hand, converting a large and busy clinical microbiology laboratory into a biosafety level III facility, where all specimens are handled in biologic safety cabinets (32), is both impractical and unnecessary, especially in laboratories in areas where the disease is not endemic. However, in regions where brucellosis is highly prevalent, an enhanced safety policy should be adopted. All blood culture vials detected as positive by the automated blood culture system, as well as all bone marrow and synovial fluid specimens, should be manipulated in biologic safety hoods until the isolated microorganisms are definitively determined to be other than *Brucella* sp (27).

For laboratories serving areas where brucellosis is uncommon, the following recommendations are made. 1) Because of the low incidence of brucellosis, physicians in areas not endemic for the disease are unfamiliar with the clinical and epidemiologic features of human brucellosis, and the possibility of brucellosis is rarely considered in the differential diagnosis. Periodic education of physicians on this subject is indicated. 2) Communication between attending physicians and the laboratory should be improved. The clinical microbiology laboratory should be informed in advance when clinical specimens had been obtained from patients with risk factors for brucellosis, such as recent history of travel to brucellosis-endemic areas, consumption of local or imported unpasteurized dairy products, or professional exposures in veterinarians, shepherds, slaughterhouse employees, and laboratory workers. 3) The use of automated, continuous monitoring blood culture systems for patients with suspected brucellosis should be preferred over the lysis-centrifugation method because the latter involves centrifuging clinical specimens and visually inspecting plates to detect the organism and probably increases the risk for transmission (11,27). Although the use of blood lysis-based methods has been advocated in the past for improving detection of *Brucella* bacteremia, modern automated blood culture systems are faster and more sensitive (11,12). 4) The familiarity of laboratory technicians with the characteristics of the organism, as well as with the safe handling of cultures, should be improved and maintained through periodic education. The reader is referred to the excellent review by Gilligan and York for handling and identifying presumptive *Brucella* organisms (35). 5) Standard precautions and strict adherence to good laboratory practices must be completely adopted, reinforced, and regularly monitored. 6) All work with gram-negative or gram-variable small rods or coccobacilli isolated from tissues, blood, bone marrow, bone, or synovial fluid exudates should be carried out in a biosafety cabinet until *Brucella* has been ruled out. 7) Plates should be sealed for safety when not in use and appropriately disposed and sterilized as soon as they are no longer being actively used (17). 8) Because brucellae are relatively slow-growing bacteria, cultures for the organism have been traditionally kept for several weeks. However, modern blood culture systems enable brucellae to be detected within the routine 5-day incubation period instituted in most clinical laboratories (12). Therefore, safety precautions should not be limited to organisms that tend to grow slowly (8,18,20,26). 9) Antimicrobial drug–susceptibility testing of *Brucella* organisms is not indicated because the therapeutic regimen for brucellosis is standard, and the organism does not usually acquire antimicrobial resistance. Performance of this and other unnecessary tests, and especially of laboratory procedures known to produce aerosols, should be strongly discouraged (27). 10) If a suspension of living brucellae is spilled and the organism is recognized, the entire laboratory should be immediately evacuated, doors should be shut, and an effective germicide such as 3% phenol or 10% bleach should be applied by a trained person wearing a safety mask, goggles, an impermeable laboratory gown, and gloves (7).

### Postexposure Prophylaxis

Because of ethical considerations, the heterogeneous nature of the events leading to *Brucella* exposure, difficulties in determining the actual risk for individual workers, late recognition of outbreaks, and the small number of persons involved in each outbreak, no controlled studies have been performed to assess the value of administering postexposure prophylaxis to persons at risk. However, anecdotal evidence suggests that administering prophylactic antimicrobial drug therapy may reduce the risk of developing clinical disease (7,8,26).

In a recently reported event, an isolate from a chest wall exudate culture from an Indian patient, which was originally identified as *M. phenylpyruvica*, was correctly recognized as *B. melitensis* 22 days after the specimen was obtained (8). By that time, 26 laboratory workers had been potentially exposed, and 6 had actually manipulated the organism. These 6 workers were considered to be at high risk and offered a 3-week prophylactic course of combined doxycycline-rifampin or trimethoprim-sulfamethoxazole therapy. None of the 5 laboratory technologists who received postexposure antimicrobial drugs became ill or developed an antibody response. However, *Brucella* bacteremia developed in the only laboratory worker who refused therapy, and she seroconverted 10 weeks after the specimen was received (8). Neither clinical disease nor seroconversion developed in the remaining 19 laboratory workers who were only present in the laboratory but were not in direct contact with the organism (8).

In another event, 3 laboratory technologists who had inadvertently worked with a *B. melitensis* isolate on an open bench and sniffed the plates were given 1 week of prophylactic doxycycline within 24 hours of the exposure; none of them became ill or seroconverted (26). In a large exposure caused by breakage of a tube containing *B. abortus*, patients who started therapy immediately after seroconversion was detected and before symptoms developed (10 weeks after the laboratory accident) had a benign clinical course, suggesting that even if the disease was not prevented, a certain degree of attenuation probably occurred (7).

Because of the high attack rate of brucellosis among exposed workers, the unpredictable and often chronic course of the disease, and the difficulties in eradicating the organism once a symptomatic infection has been established, postexposure prophylaxis is probably indicated for all persons after an obvious exposure to living brucellae (26). Antimicrobial drug prophylaxis with a combination of oral doxycycline 100 mg twice a day plus rifampin 600 mg once daily for 3 weeks should be started as soon as the exposure to confirmed *Brucella* organisms is recognized (8). For pregnant women, administration of trimethoprim-sulfamethoxazole 160/800 mg 2 × per day for 3 weeks has been advocated (8).

### Postexposure Follow-up

Whether exposed persons have received prophylactic therapy or not, increased surveillance for clinical signs of disease should be conducted for at least 6 months. In addition, exposed laboratory workers should be followed for possible subclinical infections or early signs of disease by periodic serologic testing. Weekly or semiweekly serologic surveillance is recommended for the first 3 months and once a month thereafter for 3 to 9 additional months to detect late infections resulting from prolonged incubation (7,8).

Human brucellosis has variable clinical manifestations and may mimic other infections, particularly influenza, as well as noninfectious conditions. Therefore, the diagnosis of the disease requires a high index of suspicion. Education of exposed personnel on the symptoms of the disease and the need for periodic and timely serologic follow-up are particularly important. Increased vigilance during the flu season employing a broad case definition is clearly needed, and administration of influenza vaccine should be strongly recommended to all *Brucella*-exposed persons. If symptoms develop, blood cultures and cultures of other normally sterile body fluids, as clinically indicated, should be obtained, and a new antibody titer should be determined. Although use of nucleic acid amplification methods has been shown to enable early detection of infected persons (38), these tests are not yet commercially available.

### Treating Infected Persons and Posttreatment Follow-up

Persons in whom culture-confirmed or serologically proven disease develops should receive therapy with oral doxycycline and rifampin for 6 weeks, or a combination of oral doxycycline for 6 weeks and an intramuscular aminoglycoside (gentamicin or streptomycin) for the first 2 weeks (2,7). Use of 3 drugs is usually reserved for complicated cases or life-threatening clinical manifestations, such as endocarditis or meningitis (1). Pregnant women should receive trimethoprim-sulfamethoxazole 160/800 mg 2 × per day for 6 weeks (1).

Even when patients are appropriately treated, the risk for relapse remains high (≈20%) (39,40). Therefore, patients who have completed a full therapeutic antimicrobial course should be followed clinically and serologically for 1 year. Failure to show declining antibody titers may indicate incomplete cure. If symptoms consistent with brucellosis develop, blood cultures and serologic tests should be performed to detect relapses of the disease (39). Use of nucleic acid amplification methods in the future may further improve the detection of patients in whom the organism was not eliminated (40).

Although human brucellosis has been eradicated from most industrialized countries, isolated cases of disease, usually related to travel or import of contaminated food from disease-endemic areas, continue to occur. Because of the low incidence of disease in industrialized countries, clinical laboratory technologists have become unfamiliar with identifying and handling *Brucella* species. Unsafe laboratory practices while manipulating *Brucella* isolates have resulted in inadvertent exposure to the organism and many cases of laboratory-acquired disease, indicating lack of preparedness to cope with bioterrorism threats involving brucellae. Education of laboratory personnel on the identification of *Brucella* species, adherence to and enforcement of standard precautions, thorough investigation of laboratory exposures, administration of prophylactic antimicrobial drug therapy, and close follow-up of directly exposed persons are strongly recommended.
