# Correction, Vol. 11, No. 8

**DOI:** 10.3201/eid1111.C11111

**Published:** 2005-11

**Keywords:** Errata, Erratum, Correction, Vol. 11, No. 8

In "Laboratory Exposures to Brucellae and Implications for Bioterrorism" by Pablo Yagupsky and Ellen Jo Baron, an error occurred in the dosage for rifampin prophylaxis. In the second sentence of the fourth paragraph under the heading "Postexposure Prophylaxis," the correct dosage of rifampin is 600 mg once daily.

The corrected text appears in the updated article at http://wwwnc.cdc.gov/eid/article/11/8/04-1197_article.htm.

We regret any confusion this error may have caused.

